# Supplementary figures and images for: Age-related changes in the neuromuscular control of forward and backward locomotion
Source: PLoS One. 2021 Feb 17;16(2):e0246372. doi: 10.1371/journal.pone.0246372 (PMC7888655; doi:10.1371/journal.pone.0246372)

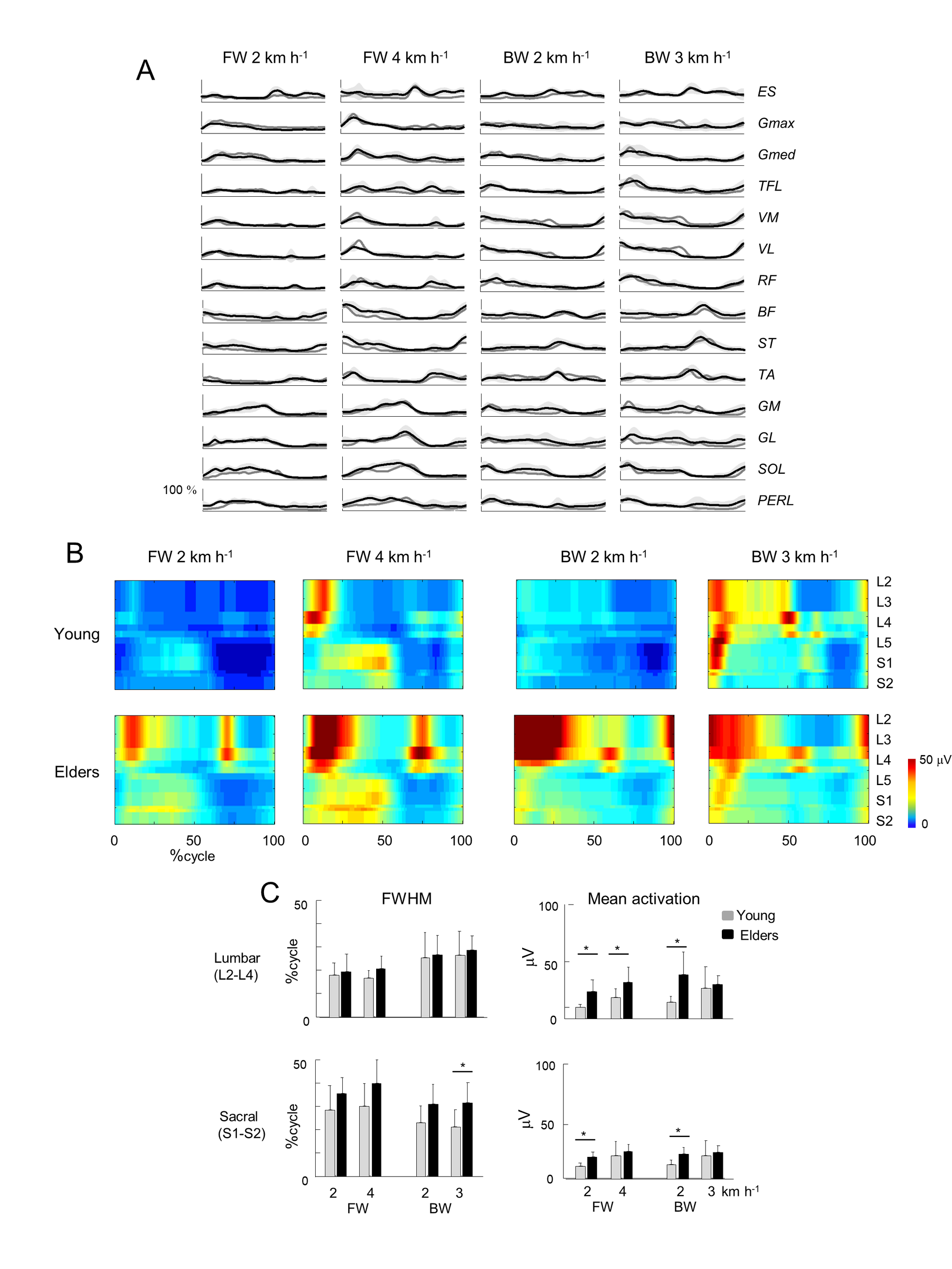

Supplement: S1 Fig — A—Ensemble-averaged normalized electromyogram (EMG) patterns. For each individual, EMG signals from each muscle were normalized to unit variance across all trials. B–Motor output (reported in μV) is plotted as a function of gait cycle in young (top) and older (bottom) adults. C–Average full width half maximum and mean activation of the lumbar (top) and sacral (bottom) segments. The bars represent the grand mean of all the young (grey) and the older (black) adults. Thin lines represent one standard deviation. The * indicates a significant effect of age. (TIF) [file pone.0246372.s001.tif]
