# Supplementary material for: Age-related changes in the neuromuscular control of forward and backward locomotion
Source: PLoS One. 2021 Feb 17;16(2):e0246372. doi: 10.1371/journal.pone.0246372 (PMC7888655; doi:10.1371/journal.pone.0246372)
Supplement: S1 Table — List of muscles analysed (1) or removed (0) for each condition (from left to right: Forward 2 km h-1; Forward 4 km h-1; Backward 2 km h-1; Backward 3 km h-1) in young (Y) and older (O) adults. (DOCX) [file pone.0246372.s002.docx]

**Table S1 –** List of muscles analysed (1) or removed (0) for each condition (from left to right: Forward 2 km h^-1^; Forward 4 km h^-1^; Backward 2 km h^-1^; Backward 3 km h^-1^) in young (Y) and older (O) adults

|  | **Y1** | **Y2** | **Y3** | **Y4** | **Y5** | **Y6** | **Y7** | **Y8** | **Y9** | **Y10** |
| --- | --- | --- | --- | --- | --- | --- | --- | --- | --- | --- |
| **ES** | 1111 | 1111 | 1111 | 1111 | 1111 | 1101 | 1111 | 0000 | 0001 | 0101 |
| **Gmax** | 1111 | 1111 | 1111 | 1100 | 1111 | 1100 | 1011 | 1111 | 0101 | 0101 |
| **Gmed** | 1111 | 1111 | 1111 | 0011 | 1111 | 1111 | 1011 | 1111 | 0101 | 0101 |
| **TFL** | 1111 | 1111 | 1111 | 1111 | 1111 | 1111 | 1111 | 1111 | 0101 | 0101 |
| **VM** | 1111 | 1111 | 1111 | 0110 | 0110 | 0011 | 1111 | 1111 | 0101 | 0101 |
| **VL** | 1111 | 1111 | 1111 | 1101 | 1101 | 1111 | 1111 | 1111 | 0101 | 0101 |
| **RF** | 1111 | 1111 | 1111 | 0110 | 1111 | 1111 | 1111 | 1111 | 0101 | 0101 |
| **BF** | 1111 | 0111 | 1111 | 1011 | 1111 | 1111 | 1111 | 1111 | 0101 | 0101 |
| **ST** | 1111 | 1111 | 1111 | 0101 | 1111 | 1111 | 1000 | 1111 | 0101 | 0101 |
| **TA** | 1111 | 1111 | 1111 | 1100 | 1111 | 1111 | 1101 | 1111 | 0101 | 0101 |
| **GM** | 1111 | 1111 | 1111 | 1111 | 1111 | 1101 | 1111 | 1111 | 0101 | 0101 |
| **GL** | 1111 | 1111 | 1100 | 1111 | 1111 | 1111 | 1111 | 1111 | 0101 | 0101 |
| **SOL** | 1100 | 1111 | 1111 | 0000 | 1111 | 1111 | 1111 | 1111 | 0101 | 0101 |
| **PERL** | 1101 | 1111 | 1111 | 0000 | 1011 | 0011 | 1111 | 1111 | 0101 | 0101 |
|  |  |  |  |  |  |  |  |  |  |  |
|  | **O1** | **O2** | **O3** | **O4** | **O5** | **O6** | **O7** | **O8** | **O9** | **O10** |
| **ES** | 1111 | 1111 | 0111 | 1111 | 1111 | 0111 | 1111 | 1110 | 1111 | 1110 |
| **Gmax** | 1111 | 1111 | 1111 | 1111 | 1111 | 0011 | 0000 | 1111 | 1111 | 1110 |
| **Gmed** | 1111 | 1111 | 1111 | 1111 | 1111 | 1111 | 0011 | 1110 | 1111 | 1110 |
| **TFL** | 0000 | 1111 | 1101 | 1111 | 1111 | 1011 | 1011 | 1111 | 1111 | 1110 |
| **VM** | 0000 | 1111 | 1111 | 1111 | 1111 | 0000 | 0000 | 1111 | 1111 | 0000 |
| **VL** | 1000 | 1111 | 1111 | 1111 | 1111 | 0000 | 1111 | 1111 | 1111 | 1110 |
| **RF** | 1111 | 1111 | 1011 | 0000 | 1111 | 0011 | 1111 | 1100 | 1111 | 1110 |
| **BF** | 1111 | 1111 | 1101 | 1111 | 1111 | 0110 | 1111 | 1111 | 1111 | 1000 |
| **ST** | 1111 | 1111 | 1111 | 1111 | 1111 | 1110 | 1110 | 1111 | 0000 | 1110 |
| **TA** | 1111 | 1111 | 1100 | 1111 | 1111 | 1011 | 1111 | 1111 | 1011 | 1110 |
| **GM** | 1111 | 1111 | 1111 | 1111 | 1111 | 1111 | 1111 | 1010 | 1111 | 1110 |
| **GL** | 1111 | 1111 | 1111 | 1111 | 1011 | 0111 | 0011 | 1111 | 1111 | 1010 |
| **SOL** | 1111 | 1111 | 1111 | 1111 | 1111 | 1111 | 1111 | 1111 | 1111 | 1110 |
| **PERL** | 1111 | 1111 | 1111 | 1111 | 1111 | 1111 | 0111 | 1111 | 1111 | 1010 |

Muscle analysed: *erector spinae* (*ES*) at L2 level, *gluteus maximus* (*Gmax*), *gluteus medius* (*Gmed*), *tensor fasciae latae* (*TFL*), *vastus medialis* (*VM*), *vastus lateralis* (*VL*), *rectus femoris* (*RF*), long head of the *biceps* *femoris,* (*BF*), *semitendinosus* (*ST*), *tibialis anterior* (*TA*), *medial gastrocnemius* (*MG*), *lateral gastrocnemius* (*LG*), *soleus* (*SOL*) and *peroneus longus* (*PERL*).
