# Supplementary material for: Age-related changes in the neuromuscular control of forward and backward locomotion
Source: PLoS One. 2021 Feb 17;16(2):e0246372. doi: 10.1371/journal.pone.0246372 (PMC7888655; doi:10.1371/journal.pone.0246372)
Supplement: S2 Table — (DOCX) [file pone.0246372.s003.docx]

**Table S2 –** Average (mean± SD) coefficient of correlation *r* between the activation of each individual spinal segment (from L2 to S2) reconstructed from a subset of seven muscles (minimum number of muscle recorded) and from the full set of muscles in each walking condition.

| Segments | Forward 2 | Forward 4 | Backward 2 | Backward 3 |
| --- | --- | --- | --- | --- |
| **L2** | 0.95±0.05 | 0.91±0.08 | 0.94±0.06 | 0.90±0.09 |
| **L3** | 0.98±0.02 | 0.97±0.03 | 0.97±0.02 | 0.96±0.05 |
| **L4** | 0.93±0.08 | 0.93±0.05 | 0.93±0.05 | 0.90±0.12 |
| **L5** | 0.93±0.14 | 0.96±0.04 | 0.96±0.05 | 0.94±0.05 |
| **S1** | 0.99±0.01 | 0.99±0.02 | 0.99±0.018 | 0.99±0.01 |
| **S2** | 0.99±0.02 | 0.99±0.02 | 0.99±0.018 | 0.99±0.01 |
